# Community pharmacists’ counseling practices and patient experiences about topical corticosteroids – an online survey in the Klang Valley, Malaysia

**DOI:** 10.1186/s12875-022-01871-z

**Published:** 2022-10-15

**Authors:** Abigail Dayang Nathan, Pathiyil Ravi Shankar, Chandrashekhar T Sreeramareddy

**Affiliations:** 1 School of Postgraduate Studies, International Medical University, Kuala Lumpur, Malaysia; 2 IMU Centre for Education, International Medical University, Kuala Lumpur, Malaysia; 3 Department of Community Medicine, International Medical University, Kuala Lumpur, Malaysia

**Keywords:** Community pharmacist, Counseling, Topical corticosteroid

## Abstract

**Background:**

Community Pharmacists (CPs) play an important role in patient counseling regarding the use of topical corticosteroids (TCS). We assessed CP’s self-reported counseling practices regarding TCS and patients’ reported counseling experiences.

**Methods:**

A previously developed questionnaire was adapted to the Malaysian context. A random sample of 364 registered CPs practicing in three states, Selangor, Kuala Lumpur, and Putrajaya were invited for an online survey. The questionnaire for CPs explored their perceived patient knowledge about topical corticosteroid (TCS) use, their counseling practices, and perceived barriers to counseling. Thirty patients were also selected from five pharmacies i.e., six consecutive patients who consulted each CP were invited to participate in the patient survey by completing a checklist about their experiences regarding the counseling received.

**Results:**

A majority (> 90%) of the CPs mostly explained to the patients that the medication was TCS and the frequency and duration of application but only 10% correctly identified scenarios needing medical referral. Only about half of the CPs always explained about side effects, strength, efficacy, and storage of TCS. The two main barriers were patients’ negative perception of TCS (65.4%) and pharmacists’ lack of time for counseling (49.7%). Counseling practice score was associated with CPs’ age (aOR 0.86, 95%CI 0.78–0.94), pharmacists’ recommendation on TCS use (aOR 0.11, 95%CI 0.02–0.61), and time spent on counseling (aOR 1.42, 95%CI 1.13–1.64). Patients mentioned they were counselled on the frequency and duration of application of TCS, and potential adverse effects. Most were not counselled on action to take when an adverse event occurs and the storage and use of leftover medication.

**Conclusion:**

CPs counseling practices to their patient about the use of TCS requires improvement. Continuing education and hands-on training are needed for CPs regarding counseling about TCS use.

**Supplementary Information:**

The online version contains supplementary material available at 10.1186/s12875-022-01871-z.

## Background

Community pharmacists (CPs), also known as retail pharmacists, play a significant role in primary health care delivery. They provide services such as supplying medication, providing medication reviews, medical and lifestyle counseling. Studies have demonstrated that utilizing CPs for the treatment of minor ailments results in optimizing health resources and reducing congestion at primary health facilities [1]. Services provided by CPs have been shown to improve patient care and health outcomes [2, 3]. Strand et al. (2014). posit that pharmacists have the potential to contribute to and enhance population health [4]. The COVID-19 pandemic has seen greater authority granted to pharmacists in many developed nations. Pharmacists have been authorized to prepare disinfectants, renew chronic treatment prescriptions, perform screening tests, and administer vaccines. Countries have also facilitated virtual consultations and home delivery of medicines. [5] In Malaysia, in addition to providing general medication counseling and health promotion services, CPs are also authorized to supply non-prescription Group C poisons to consumers as per the Poison Act 1952 [6]. Examples of group C poisons include preparations of homatropine, hyoscine, aminophylline, bromhexine, cortisone and hydrocortisone topical preparations among others.

The first schedule to the poisons list classifies medicines into part 1, part 2, and exempt categories; type 1 is further subdivided into groups A, B, C, and D [7]. Topical corticosteroids (TCS) are included under Group C poisons, making it a common medication prescribed and sold in private healthcare facilities, including community pharmacies [8]. During the counseling session, the frequency and dosage of application should be communicated effectively, for example, TCS is applied as a thin layer once or twice a day, depending on potency and formulation, and the dosage is expressed as a ‘fingertip unit’[9]. A finger-tip unit (FTU) is the amount of ointment expressed from a tube with a 5 mm diameter nozzle, applied from the distal skin-crease to the tip of the index finger [10].

Current clinical practice guidelines recommend the use of TCS as an adjunct with other treatments such as emollients, antibiotics, or antifungal topical medications, as well as systemic therapy with antibiotics, antihistamines, or oral steroids where appropriate [11]. Improper use of TCS can aggravate an underlying superficial fungal or bacterial infection of the skin and prolonged exposure may result in skin thinning, striae, hyper- or hypopigmentation, hirsutism, and short stature in children or Cushing’s syndrome [12]. Therefore, correct information provided to the patients about TCS is very important and CPs have an important role to play when they dispense TCS to patients at pharmacies.

CPs have the knowledge to advise patients and caregivers on optimum TCS use, and the provision of optimum and good quality counseling by CPs is associated with better treatment outcomes [13]. The key factors associated with the quality of counseling and the role CPs play in encouraging high-quality counseling practices are vital to ensure patients’ safe and effective use of TCS. A previous study conducted in Portugal identified that less than 18.2% of patients received information on dosage, frequency, and duration of application of topical medication [14]. While studies have been conducted on the use of topical medications among patients with psoriasis [15] and on pharmacists’ knowledge about the use of topical antibacterials in Malaysia [16], the counseling practices of TCS have previously not been studied. We aimed to assess the counseling practices of the community pharmacists regarding the use of TCS and determine the factors associated with counseling practices. We also studied patient experience with counseling received from CP about TCS to corroborate the counseling practices reported by the CPs.

## Methods

### Design, setting, and participants

A cross-sectional online survey was conducted among community pharmacists practicing in three Malaysian states (Selangor state, and the federal territories of Kuala Lumpur, and Putrajaya). Community pharmacists holding Type A license certification and currently working in community pharmacies were involved. Type A license is issued by the pharmacy enforcement at the state level and allows a pharmacist to import, store and deal generally with all poisons by wholesale and retail or by wholesale only or by retail only [17].

### Study size

The sample size for CPs was determined using the formula for the finite population. The expected proportion was 0.5. The proportion of CPs who had correct knowledge and practiced correct counseling techniques was not available from the literature. For a confidence limit of 95% and 5% allowable error, the calculated minimum sample size was 303. Taking into consideration an anticipated non-response rate of 20%, the final sample size was 364.

### Sampling methods

A list of 1,207 CPs holding licenses from Selangor and 601 from Kuala Lumpur and Putrajaya was obtained from the Official Portal of the Pharmaceutical Services Programme [18]. Names and types of licenses were filtered to verify addresses, eliminate duplicates, and exclude those employed in private hospitals, compounding pharmacies, and veterinary pharmacies. After screening, a final list of community pharmacists holding type A license was created. The list had 925 CPs from Selangor and 473 from Kuala Lumpur and Putrajaya. From this list, the CPs were selected for the survey by systematic sampling. Of the required sample size of 364 participants, 60% were from Selangor (218) and the remaining 40% were from Kuala Lumpur and Putrajaya (146). Sampling interval (sample required/total sample of CPs) was approximately four (925/218 = 4.2) for Selangor and (473/146 = 3.3) three for Kuala Lumpur and Putrajaya.

### Questionnaire

The questionnaire for CPs was adapted with permission from the original questionnaire developed by Kang et al. [13]. The questionnaire (appendix 1) consisted of four sections. Section A collected information about socio-demographic characteristics of pharmacists, dispensing and sales of TCS in their pharmacy. Section B focused on type, and time taken to counsel and the extent to which various aspects of TCS use were covered and the respondent’s perceived barriers to counselling. Section C covered questions about CPs perception of the patient’s knowledge on TCS prior to the counselling. Section D assessed CPs knowledge about adverse drug events related to TCS. The original questionnaire was modified to suit local product formulations and dispensing practices. Other modifications such as questions on ethnicity and options for current workplace location were altered. The questionnaire was pilot tested among 25 participants who were recruited using purposeful and snowball sampling. The Cronbach’s alpha value obtained after the pilot test was 0.69.

### Variables

For multiple linear regression analyses., the independent variables were socio-demographic characteristics as age, gender, work location, work status, years of experience as a community pharmacist, and education level. Dependent variables were CP’s perception regarding patients’ knowledge about TCS measured using eight “yes/ no” type questions. The score for CPs perceived patient’s knowledge of TCS was obtained by adding the responses to eight items. The response options ‘yes’ or ‘no’ were coded as ‘1’ and ‘0’ respectively. The score on CPs counselling practices on TCS use was measured using an 11-item, 3-point Likert scale (explain most of the time, explain half the time and did not explain most of the time scored as ‘2’, ‘1’, and ‘0’ respectively). The sum of scores represents the counselling practice scores (maximum score of 22).

### Data Collection

Due to the Coronavirus (COVID-19) pandemic, an online questionnaire was distributed from September to December 2021. Selected pharmacists were contacted using the email addresses or premise phone numbers listed on the pharmaceutical services website or obtained following a Google search, or via social media platforms such as Facebook or LinkedIn. After establishing contact with participants, the information sheet and questionnaire were delivered through email, or a link supplied via WhatsApp, Facebook, or LinkedIn messages. Participants who completed the questionnaire received RM5 (approx. USD 1.25) e-vouchers as a token of appreciation.

### Patient study

CPs who participated in the online survey were requested to recruit patients who came to purchase TCS. Five of the CPs agreed to recruit patients for the sub study among the patients. From each of the five selected pharmacies, six consecutive consenting patients were selected. The questionnaire for patients was extracted and modified from Question 12 in the original Kang et al. questionnaire [13]. The questionnaire comprised of six items about if the patient had received a specific component of counselling during the consultation with the CP. The response options were ‘yes’ or ‘no’ questions (appendix 2). Modifications were made to ensure layperson terms were used for the benefit of patients answering the questionnaire. The English language survey was also translated into the Malay language following standard recommended practices.

Patients visiting the selected community pharmacy to purchase a TCS were invited to answer a self-administered anonymous online survey if they were able to read English or Malay language. The pharmacist explained the study details to the patients and obtained their consent. The patients were instructed to scan a QR code using their smartphones to access the study information sheet followed by an online survey about their perceptions regarding counselling received from the CPs about the use of topical corticosteroids. The participants were instructed to complete the survey after leaving the pharmacy premises.

### Data analysis

IBM SPSS (version 25) was used to analyse the data. Descriptive statistics were calculated, and the distribution of scores were checked for normality. Bivariate comparisons of CPs counselling practice score on TCS use were done with demographic factors of CPs, time taken for preparation and conduct of counselling on TCS use, perceived barriers to counselling and delivery time, training about adverse drug events (ADE) and the action taken if ADE to TCS was experienced by the patient. The bivariate comparisons that were significant at p < 0.25 were included in the multivariate analyses. CPs perceived patient knowledge of TCS scores, and TCS counselling practice scores were the outcome variables. Multiple linear regression analysis was conducted to identify the factors associated with the CPs counselling practice score. Adjusted odds ratios and the 95% confidence interval (CI) were estimated (p < 0.05).

The International Medical University Joint Committee for Research and Ethics approved this research (Project ID number: MSPH I/2021(04)) on the 14th of February 2021. Informed consent was obtained from the CPs participating in the survey and the procedures were followed as per the declaration of Helsinki.

## Results

### Response rates and characteristics of CPs

The questionnaire were sent to 364 participants, out of whom 215 completed the questionnaire. The response rate was 70% and all responses were complete. Table 1 shows demographic and other characteristics of the CPs. Out of 215 respondents who completed the survey, 155 (72.1%) were females and 60 (27.9%) were males. The median age of participants was 29 years (IQR 28–31), with a median of 3 (2–5) years of experience as a community pharmacist. Most participants were of Chinese ethnicity (70.7%), worked in chain pharmacies (65.5%), were full-time employees (87.0%), had a bachelor’s degree (94.4%), and indicated that they had been trained in ADEs (82.3%) (Table 1).

**Table 1:** Demographic characteristics of the community pharmacists who participated in the study

Variable	Frequency, N = 215	%
**Age (median and IQR years)**	29.0 (28–31)	
**Gender**		
Male	60	27.9
Female	155	72.1
**Ethnicity**		
Chinese	152	70.7
Malay	26	12.1
Others	37	17.2
**Pharmacy location**		
Selangor	126	58.6
Kuala Lumpur and Putrajaya	89	41.4
**Type of Pharmacy**		
Pharmacy chain	141	65.5
Independent Pharmacy	74	34.4
**Work Status**		
Full-time	187	87.0
Part-time	28	13.0
**Education level**		
Degree	203	94.4
Post-graduate	12	5.6
**Trained in adverse drug events (ADE)**		
Yes	177	82.3
No	38	17.7

### CPs practices related to TCS counseling

About 97% of the CPs correctly identified “14 days or less” as the maximum duration for TCS treatment and 85.6% of the CPs correctly ranked three TCS in decreasing order of their potency. About 1 in 10 CPss10.2%) accurately identified scenarios that necessitate medical referrals. About 98% of CPs perceived that “pharmacist’s explanation” and 90.2% perceived “internet” as patient’s main source of information on TCS. Respondents rated steroid-only TCS as the most sold compared to other steroid-combination products, followed by steroid-antibiotics, steroid-antifungals, steroid-keratolytic, and steroid-other medicines combinations. Pharmacists ranked medication misuse and medication characteristics, such as potency of TCS, as the most common cause of adverse drug events; followed by patient characteristics, (patient’s age), and medication overuse. Dry skin and skin irritation were the most reported adverse drug events, followed by skin discolouration and skin infections. When patients contacted community pharmacists about adverse drug events caused by topical corticosteroids, 79.5% recommended them to discontinue the medication and advised them to see a doctor. Additionally, 76.7% of respondents indicated they would recommend treatment re-trial following a medication review and patient re-education. Only 27.0% of participants would report the event to the National Pharmaceutical Regulatory Agency (NPRA).

### Dispensing characteristics of TCS, counselling about TCS use and barriers

**Table 2:** CP reported monthly sales, dispensing practices, and barriers to counseling

**Dispensing and sales of TCS (monthly average)**	**Median (IQR)**
Non-prescription TCS (%)	60.0 (30.0–90.0)
Pharmacist’s recommendation (%)	50.0 (30.0–70.0)
Counseling preparation time (minutes)	5.0 (5.0–8.0)
Time spent counseling for each prescription TCS (minutes)	5.0 (4.0–10.0)
Time spent counseling for each non-prescription TCS (minutes)	5.0 (3.0–8.0)
**Barriers**	**Frequency (%)**
Patient’s negative perception of topical corticosteroids	100 (65.4)
Lack of time for counseling	76 (49.7
Lack of counseling material	62 (40.5)
Presume patients already know about topical corticosteroids	47 (30.7)
Doctor’s negative perceptions of pharmacists’ counseling	21 (13.7)

Table 2 shows dispensing characteristics and barriers to counselling about TCS. The CPs estimated that 60% (IQR 30.0–90.0) of TCS sold were non-prescription, with half (50%, IQR 30.0–70.0) sold on pharmacist’s recommendation. The CPs estimated a median time of 5.0min each to prepare for counselling and to counsel the patient The frequency of explaining to the patient about various aspects of TCS use are shown in Table 3. The proportion of CPs who reported that they provided information on the medication being TCS, frequency of use and duration of use was more than 86%. However, < 50% of the CPs reported that they provided information about potency, method of application, strength (dose) and side effects (Table 3).A total of 153 of 215 respondents reported at least two barriers to counseling. Among 306 responses received patient’s negative perception towards TCS (65.4%), lack of time for counselling (49.7%), lack of counselling material (40.5%), and presuming patients already knew about TCS (30.7%) were commonly selected barriers by the CPs (Table 2).

**Table 3:** Frequency of explaining the TCS use by the CPs and barriers to counseling

Counseling aspects of TCS use	Most of the time	Half the time
How to use- **frequency** of application in a day	92.1	7.0
That it is a topical corticosteroid	90.2	7.9
How to use- **duration** of treatment	86.5	11.6
How to use- **choice of formulation** (ointment, cream, lotion, etc.)	50.7	33.0
Adverse drug events	49.8	41.4
Skin conditions and diseases where TCS should not be used	47.0	38.1
Expected efficacy and effectiveness	44.7	47.4
Strength (potency)	44.7	44.7
Symptoms to look out for in an adverse drug event	19.5	47.9
How to use- dosage	55.3	32.1
Precautions for storage and application of leftover topical corticosteroids after treatment completion	13.0	36.3

### Factors associated with counselling practice score

Table 4 shows the bivariate comparison of CPs counselling practice scores with demographic factors and factors related to counselling about TCS. The scores were higher among younger CPs, those trained in ADE reporting, those who spent more time preparing for counselling and counselling patients, those who perceived greater barriers to counselling and those with a higher CPs perceived patient’s knowledge score. Counselling practice score was associated with age, training on adverse dug events, time spent on counselling, CPs perceived patient’s knowledge, and perceived barriers (< 0.05). On multivariate analyses, counselling practice score decreased by a factor of 0.86 for each one-unit increase in age (p = 0.001). Practice score decreased by a factor of 0.11 for each one-unit increase in percentage of TCS supply on pharmacists’ recommendation (p = 0.037). Counselling practice scores increased by a factor of 1.42 (p = < 0.001) for each unit increase in time spent on counselling (Table 5).

**Table 4:** Bivariate comparison of the CP counseling practice score on TCS use with demographic, practice-related factors

	Mean practice score (SD)	Correlation coefficient	p-value
**Age (years)** ^**€**^		-0.18	0.009*
**Sex** ^**¶**^			
Male	14.72 (3.27)		0.062
Female	15.63 (3.15)		
**Ethnicity** ^**¥**^			
Chinese	15.15 (3.38)		0.145
Malay	15.39 (2.80)		
Others	16.30 (2.56)		
**Pharmacy location** ^**¶**^			
Selangor	15.22 (3.29)		0.415
Kuala Lumpur & Putrajaya	15.58 (3.08)		
**Type of Pharmacy** ^**¶**^			
Pharmacy chain	15.46 (3.11)		0.575
Independent Pharmacy	15.20 (0.39)		
**Work status** ^**¶**^	15.44 (3.27)		
Part-time	14.89 (2.70)		0.397
Full-time	15.44 (3.27)		
**Experience as a community Pharmacist (years)** ^**€**^		-0.09	0.188
**Education** ^**¶**^			
Degree	15.45 (3.13)		0.287
Post-graduate	14.08 (4.17)		
**Trained** **in ADE** ^**¶**^			
No	14.10 (3.70)		0.007
Yes	15.64 (3.03)		
**Supply of TCS by pharmacist’s recommendation (%)** ^€^		0.14	0.037
**Counseling preparation time (mins)** ^€^		0.27	< 0.001
**Time spent counseling about prescription TCS (mins)** ^€^		0.38	< 0.001
**Time spent counseling about non-prescription TCS (mins)** ^€^		0.32	< 0.001
**Perceived barriers to counselling** ^**¶**^			
No	14.40 (3.47)		0.004
Yes	15.77 (3.01)		
**Experienced any patients’ who reported ADE** ^**¶**^			
No	15.40 (3.31)		0.838
Yes	15.30 (2.90)		
**Knowledge score** ^**§**^		0.14	0.043

^¶^ Independent Samples t-test, ^¥^ One-way analysis of variance, ^€^ Spearman’s rank correlation coefficient

**Table 5:** Multivariate analyses of factors associated with counseling practice scores on TCS use

	Adjusted Odds Ratio (95% CI)	p-value
**Age (years)**	0.86 (0.78–0.94)	0.001
**Education level**	0.11 (0.02–0.61)	0.011
**Trained in ADE**		
No	1	0.066
Yes	0.39 (0.14–1.07)	
**Supply of TCS by pharmacists recommendation**	0.11 (0.02–0.61)	0.037
**Counseling preparation time (mins)** ^**€**^	1.15 (1.01–1.33)	0.060
**Time spent counseling about prescription TCS (mins)**	1.42 (1.23–1.64)	< 0.001
**Time spent counseling about non-prescription TCS (mins)**	1.04 (0.82–1.25)	0.083
**Perceived barriers to counseling**		
No	1	0.171
Yes	1.92 (0.75–4.85)	
**Knowledge score**	1.31 (0.77–2.21)	0.318

### Patient’s experience receiving counselling from CPs

A total of 30 patient responses were obtained in the sub study among the patients to verify the counseling practices reported by the CPs. Their mean age was 43.3 years (SD = 10.9). Of the 30 patients, 19 (63.3%) were females, 15 were of Malay ethnicity while 11 (36.7%) were Chinese. Sixteen16 respondents (53.3%) were prescribed a steroid only preparation while 12 were prescribed a combination of a steroid and antimicrobial. Twenty (66.7%) purchased TCS for their own use. The content of counselling received by patients from a CP was also surveyed (Table 6). Patients were asked if they received specific counselling about their purchased medication from a pharmacist, such as skin conditions that the medication should not be used on, potency, dosage, frequency, duration, and information about potential adverse events. All patients were informed of the frequency and duration of application of medication, and that the product purchased is a TCS. Most patients were also counselled on the potency of the TCS, how to apply the prescribed medication, and dosage and potential side effects. However, less than half were counselled on the appropriate action to take when an ADE occurs, and the storage and use of leftover medication.

**Table 6:** Content of counseling received by patients (N = 30)

Counseling content received	Frequency	Percentage
It is a topical corticosteroid	30	100
Skin conditions where the topical corticosteroid should not be used	20	66.7
Potency (strength)	24	80.0
Dosage	28	93.3
Frequency	30	100
Duration	30	100
Potential adverse effects of the topical corticosteroid	21	70
What to do when an adverse drug event occurs	14	46.7
Storage and use of leftover medication	11	36.7

## DISCUSSION

### Main findings

The CPs working in Klang valley were surveyed online to explore the counselling they provided to their patient seeking to purchase TCS and the factors associated with the counseling practice. CPs most often provided information that the medication was a TCS, methods of application, frequency and duration of its use which was in conformity with patient reported counseling experience. Counselling practice scores were shown to be significantly associated with age, education level of the CP and time spent preparing and delivering counselling. In the current study, CPs had sufficient knowledge to conduct patient counselling and perceived themselves to be information providers for patients. Congruent to prior research by Kang et al., there was a significant association between knowledge of the patient as perceived by the CPs and counselling practice scores [13]. Additional characteristics associated with counselling practice scores, such as age, work status, supply of TCS according to pharmacists’ recommendation, and time providing counselling were noted in this study.

### Socio-demographic characteristics and counselling practices

The age of CPs had a significant association with their counselling practice scores. Counselling practice scores decreased with increasing age. This may be anticipated, since younger pharmacists are typically recent graduates who have been exposed to a patient-centred pharmacy curriculum emphasising effective communication skills. As a result, they would have developed greater knowledge and skills to counsel patients comprehensively regarding the treatment and management of minor ailments [19]. The pharmacists participating in this study perceived patients to have good knowledge about TCS. Time spent to deliver counselling had significant association with counselling practice scores as well. These findings support a previous study’s conclusion that pharmacists who spend adequate time with patients provide better medication counselling [12]. The supply of TCS on pharmacists’ recommendation also showed significant association with counselling practices. This is consistent with the assumption that CPs may not provide exhaustive information on side effects to prevent the development of steroid phobia [20]. CPs who had been trained in ADE reporting had higher counselling practice scores. ADE reporting and counselling are part of the extended roles of CPs and receiving training in ADE reporting may also have created a positive attitude toward patient counselling.

In this study only 27% of CPs mentioned that they would report an ADE/ADR. ADR reporting is influenced by several factors. In Korea it was seen that knowledge of spontaneous reporting, prior experience of ADRs and less concern about barriers to reporting were associated with higher reporting rates [21]. A study conducted in four northern states in Malaysia found that only 12.9% of pharmacists had reported an ADR and barriers identified included lack of knowledge on how to report an ADR, non-availability of reporting forms and lack of knowledge regarding where the reports were to be sent [22]. However, the CPs had a positive attitude toward ADR reporting.

### Barriers to counseling on TCS

Participating pharmacists also reported a lack of time for counselling as a barrier to providing counselling. Research postulated that the more time a pharmacist had to advise patients on the use of TCS, the more positive were the pharmacists’ views towards information provision [21].

Most participating pharmacists reported experiencing barriers to counselling. The biggest barrier was patients’ negative perceptions of TCS, followed by the lack of time for counselling. This result is consistent with studies conducted among CPs in Korea [113]. Steroid phobia has been extensively researched in dermatological conditions, and is prevalent among both patients and caregivers [23]. The correlation between steroid phobia and the aversion to using TCS is well established, and this may lead to non-adherence, insufficient therapy, and treatment failure 20].

### Content of counseling about TCS

In this study, counselling by CPs often included information on the “expected effectiveness”, “potency”, “dosage”, and “frequency of application” regardless of their perception of patient comprehension. If the CP considered that a patient may be unfamiliar with information such as “it is a topical corticosteroid”, “action to take in case of an adverse drug event”, “duration of application”, and “storage and use of remaining medication following treatment completion”, it was provided. These findings are almost identical to those of Kang et al., who hypothesised that counselling information is impacted by pharmacists’ assumptions about patients’ knowledge rather than real patient needs [13].

Poor knowledge about the use of TCS in atopic dermatitis was noted among Australian pharmacists and these appear to be modifiable through targeted education provided by a dermatologist [24]. Better communication between pharmacists and dermatologists can ensure patients receive accurate and unified counselling on TCS according to a study conducted in Wisconsin, United States [25]. In a study conducted in Pakistan, patient characteristics, free availability of TCS, and lack of physician-pharmacist coordination were regarded as major factors promoting misuse of TCS and lack of time and of counselling materials were mentioned as significant barriers to counselling [26]. In Japan, due to the lack of adequate knowledge pharmacists provided incorrect advice about the fingertip unit as a unit of TCS dose in atopic dermatitis [27]. These studies show problems with knowledge about TCS counselling among pharmacists in different countries.

### Patient experiences of counseling received from CPs about TCS

To the best of our knowledge, this is the first study to explore patients’ experience receiving counselling from a CP on the use of TCS in Malaysia. The results show that patients’ experiences about counseling provided by CPs and CPs practices on counseling for TCS agreed with the information provided and the quality of counselling. Most patients indicated they received counselling on the potency, dosage, and frequency of the TCS purchased. This was consistent with the pharmacists’ self-reported behaviours of information provision. These results differ from a previous study by Teixeira et al. showing inconsistencies between pharmacists’ self-reported behaviour and patients’ claims where only 1 in 4 patients was informed of the dosage and 1 in 3 were provided with information about the duration of use of the medication prescribed [14]. Pharmacists are more likely to provide information about adverse effects of TCS if they perceive that patient are unaware but withhold further information to avoid the development or perpetuation of steroid phobia [13, 21].

### Limitations

This study also has several limitations. First, this study was conducted among CPs in Selangor, Kuala Lumpur, and Putrajaya only. Hence, the results may not be generalizable to all CPs in Malaysia. Larger nationwide studies on community pharmacists from urban, suburban, and rural places of practice would be beneficial. Second, this study did not evaluate the clinical outcomes of patients, to examine the influence of pharmacists’ counselling in the treatment of dermatological conditions. The third limitation was the small number of patients is non-representative of all patients receiving counselling about TCS. In a sub study 30 patients who received counseling from five CPs were surveyed to verify the TCS counseling reported by the CPs. Counselling experiences reported by the patients selected by CPs themselves is likely to be affected by the Hawthorne effect. This was a result of the strict social distancing measures during the Coronavirus pandemic. Lastly, since this was a self-administered questionnaire, social desirability and self-reported biases may impact responses. However, internal consistency of data collected and the parallels in results with earlier studies are significant, suggest that the findings are reliable.

## Conclusion

The CPs mainly counsel the patients about the frequency and duration of application of TCS. Patients sub study confirmed the CP reported counselling practices. Age of CPs, counselling preparation and delivery time were significantly associated with counselling practice. Continuing education and hands-on training and a greater collaboration between different healthcare providers are needed to improve counselling about TCS provided by the CPs.

## Electronic supplementary material

Below is the link to the electronic supplementary material.

Supplementary Material 1

Supplementary Material 2

## Data Availability

The data associated with the study is available from https://figshare.com/articles/dataset/Knowledge_and_practice_regarding_counselling_of_topical_corticosteroids_among_community_pharmacists/19352540.

## Declarations

**Ethics declarations Ethics approval and consent to participate**.

This study was approved by the International Medical University Joint Committee on Research and Ethics. We sought informed consent for participation from both the community pharmacists and patients. All the procedures for ethical approval and consent procedures were followed as per declaration of Helsinki.

## Abbreviations

ADE: Adverse Drug Events
ADR: Adverse Drug Reaction
CPS: Community Pharmacists
IQR: Inter Quartile Range
TCS: Topical Corticosteroids
