# Supplementary material for: Community pharmacists’ counseling practices and patient experiences about topical corticosteroids – an online survey in the Klang Valley, Malaysia
Source: BMC Prim Care. 2022 Oct 15;23:263. doi: 10.1186/s12875-022-01871-z (PMC9569015; doi:10.1186/s12875-022-01871-z)
Supplement: Supplementary file 1 — Supplementary Material 1 [file 12875_2022_1871_MOESM1_ESM.docx]

A questionnaire to explore community pharmacists’ personal and work characteristics and their perception regarding patient counselling of topical corticosteroids

|  | **Item** | **Information** | | **Code/ Score** |
| --- | --- | --- | --- | --- |
| **A. Demographic characteristics, dispensing and sales of topical corticosteroids** | | | | |
| **1** | Age (years) |  |  |  |
| **2** | Gender | Male |  | 1 |
|  |  | Female |  | 2 |
| **3** | Ethnicity | Chinese |  | 1 |
|  |  | Malay |  | 2 |
|  |  | Indian |  | 3 |
|  |  | Bumiputera Sabah |  | 3 |
|  |  | Bumiputera Sarawak |  | 3 |
|  |  | Others |  | 3 |
| **4** | Current workplace | Selangor |  | 1 |
|  |  | Kuala Lumpur |  | 2 |
|  |  | Putrajaya |  | 2 |
| **4a** | Type of community pharmacy | Chain pharmacy |  | 1 |
|  |  | Independent pharmacy |  | 2 |
| **4b** | Work status | Full-time (FRP) |  | 1 |
|  |  | Full-time (PRP) |  | 1 |
|  |  | Part-time (Locum) |  | 2 |
| **5** | Experience as a community pharmacist (excluding experience at hospitals and pharmaceutical companies) (years) | (years) |  |  |
| **6** | Level of education | Degree |  | 1 |
|  |  | Post-graduate |  | 2 |
| **7** | Have you ever been trained in reporting adverse drug events? | Yes |  | 1 |
|  |  | No |  | 2 |
| **8** | What is the % of total sales of non-prescription TCS at your pharmacy on average per month? | Non-prescription (%) | X |  |
|  |  | Prescription (%) | 100 − X |  |
|  |  | **Total** | **100%** |  |
| **9** | What is the % of sales by recommendation of non-prescription TCS at your pharmacy on average per month? | Patient self-selection (%) | Y |  |
|  |  | Pharmacist recommendation (%) | 100 − Y |  |
|  |  | **Total** | **100%** |  |
| **10** | Please rate the following non-prescription over-the-counter topical corticosteroids in order of sales frequency  **(Note give 1 to the most frequently sold agents and 4 to the least frequently sold)** | Steroids only agents (e.g. DermAid 1% Hydrocortisone cream, Elomet Cream 0.1%) |  |  |
|  |  | Steroid-antibiotics or steroid antifungals combination agent (e.g. Fucicort cream, Daktacort cream) |  |  |
|  |  | Steroid-keratolytic agent (e.g. Beprosalic ointment) |  |  |
|  |  | Steroid-other ingredients combination agents (e.g. Daivobet ointment) |  |  |

| **B. Patient Counselling on the Use of Topical Corticosteroids** | | | | | | | | |
| --- | --- | --- | --- | --- | --- | --- | --- | --- |
| **11** | Please answer the following questions about your patient counseling practices on the use of topical corticosteroids | | | | | | | |
| **11a** | The most frequently used counseling method | | Printed Information | | | |  |  |
|  |  |  | Face-to-face (verbal) | | | |  |  |
|  |  |  | Face-to-face (verbal and printed) | | | |  |  |
|  |  |  | Demonstration of Application | | | |  |  |
| **11b** | Counselling Preparation time  “Counselling preparation time” refers to the time invested in searching, reviewing, evaluating, and organizing information, including verifying prescription and checking patients past medical history before counselling a patient. | | On average minutes per patient | | | |  |  |
| **11c** | Time spent in face-to-face (verbal) counselling | | On average minutes per patient on **prescription** topical corticosteroids | | | |  |  |
|  |  |  | On average minutes per patient on **non-prescription** topical corticosteroids | | | |  |  |
| **12** | Please mark on the items below to indicate to what extent you counsel patients using topical corticosteroids | | | | | | | |
|  |  | **Explain most of the time (2)** | | **Explain half the time (1)** | | **Do not explain most of the time (0)** | | |
| **12a** | That it is a topical corticosteroid |  | |  | |  | | |
| **12b** | Expected efficacy and effectiveness |  | |  | |  | | |
| **12c** | Skin conditions and diseases where topical corticosteroids should not be used  E.g.: do not use on burns |  | |  | |  | | |
| **12d** | Strength (potency) |  | |  | |  | | |
| **12e** | Adverse drug events |  | |  | |  | | |
| **12f** | Symptoms to look out for in an adverse drug event |  | |  | |  | | |
| **12g** | How to use- dosage  E.g.: **fingertip unit**: amount of ointment or cream squeezed out of a tube, from the tip of an adult index finger to the first crease in the finger, which may be sufficient for a diseased site of two adult-hand size |  | |  | |  | | |
| **12h** | How to use- **frequency** of application in a day |  | |  | |  | | |
| **12i** | How to use- **duration** of treatment |  | |  | |  | | |
| **12j** | How to use- **choice of formulation** (ointment, cream, lotion, etc.) for specific application site |  | |  | |  | | |
| **12k** | Precautions for storage and application of leftover topical corticosteroids after treatment completion  E.g.: do not remove medication from its original packaging and transfer to a separate container,  do not use remaining medication in other conditions at patients’ own discretion |  | |  | |  | | |
| **Questions 13-15 are regarding your counselling practices to assist patient self-treatment with non-prescription over-the-counter topical corticosteroids** | | | | | | | | |
| **13** | In which of the following cases would you recommend a patient seeking non-prescription over-the-counter topical corticosteroids to see a doctor? (Multiple selection allowed) | | Skin lesions with effusion | |  | | | 1 |
|  |  |  | Skin conditions of moderate or greater severity | |  | | | 1 |
|  |  |  | Signs of skin infection | |  | | | 1 |
|  |  |  | Neonates and infants (less than 24 months old) (excluding nappy rash) | |  | | | 1 |
|  |  |  | Applying topical corticosteroids on a large area of body surface | |  | | | 1 |
| **14** | For how many **days** do you tell patients on non-prescription over-the-counter topical corticosteroids that it is okay to use continuously? | | Maximum days | |  | | |  |
| **15** | Do you believe that there are **barriers** to counselling patients on topical corticosteroids?  (“No”, to move on to Question 17) | | Yes | |  | | | 1 |
|  |  |  | No | |  | | | 0 |
| **15a** | Please select the barriers to counseling (multiple selection allowed) | | Lack of counselling material | |  | | |  |
|  |  |  | Lack of time for counselling | |  | | |  |
|  |  |  | Doctor’s negative perception towards pharmacists’ counselling | |  | | |  |
|  |  |  | Patients’ negative perception towards topical corticosteroids | |  | | |  |
|  |  |  | Presume patients already know well about topical corticosteroids | |  | | |  |

| **C. Pharmacists’ perception on patients’ knowledge on topical corticosteroids prior to counselling** | | | | | |
| --- | --- | --- | --- | --- | --- |
| **16** | Do you think patients have adequate knowledge on the following aspects of topical corticosteroids? | | | |  |
|  |  | | Yes | No |  |
| **16a** | That it is a topical corticosteroid | |  |  |  |
| **16b** | Efficacy and effectiveness | |  |  |  |
| **16c** | Strength (potency) | |  |  |  |
| **16d** | (Patients on **non-prescription** topical corticosteroids) Adverse drug events | |  |  |  |
| **16e** | (Patients on **prescription** topical corticosteroids) Adverse drug events | |  |  |  |
| **16f** | What to do when an adverse drug event occurs | |  |  |  |
| **16g** | Know how to use- **(dosage, treatment duration, frequency of application, choice of formulation** for specific application site) and to comply with it | |  |  |  |
| **16h** | Know how to store and apply leftover topical corticosteroids after treatment completion and comply with it  E.g. do not use remaining medication on other conditions at patients’ own discretion | |  |  |  |
| **17** | Please select **three** options from the given choices that you believe are patients’ main source of information about topical corticosteroids | Pharmacist’s explanation | |  | 1 |
|  |  | Doctor’s explanation | |  | 2 |
|  |  | Product information leaflet (PIL) | |  | 3 |
|  |  | Internet | |  | 4 |

| **D. Adverse drug events of topical corticosteroids** | | | |
| --- | --- | --- | --- |
| **18** | Have you had patients complaining (including visits and phone calls) of adverse drug events after using TCS at your pharmacy | Yes | 1 |
|  |  | No | 0 |
| **19** | Please rank the factors you think are the causes of adverse drug events of TCS  Please rank the factors from 1 to 4, give “1” to the most likely factor | Medication misuse (e.g. Use on not applicable conditions) |  |
|  |  | Medication characteristics (e.g. Strength/ potency) |  |
|  |  | Patient characteristics (e.g. Elderly, with chronic diseases, neonates and infants) |  |
|  |  | Medication overuse (e.g. Patient self-treatment, not complying with treatment instructions) |  |
| **20** | Please select three (3) adverse drug events of TCS that patients frequently complain | Dry skin, itchiness, irritation |  |
|  |  | Bruises |  |
|  |  | Acne, folliculitis |  |
|  |  | Hirsutism (excessive hair growth) |  |
|  |  | Systemic adverse events (including ocular symptoms) |  |
|  |  | Capillary dilatation |  |
|  |  | Skin atrophy, stretch marks |  |
|  |  | Chang in skin colour (decolourisation, pigmentation) |  |
|  |  | Hot flashes, rosacea, perioral dermatitis |  |
|  |  | Skin infection (bacterial, fungal, viral) |  |
| **21** | What do you do when patients on topical corticosteroid complain of adverse drug events?  Multiple selection allowed | Discontinue and recommend seeing a doctor |  |
|  |  | Report to National Pharmaceutical Regulatory Agency (NPRA) |  |
|  |  | Check if the patient has been using topical corticosteroids as directed. Re-educate patient and recommend re-trial of the treatment |  |
| **22** | Please rank the below three topical corticosteroids according to their strength (potency)  Rank TCS strength (potency) from 1-3, give "1" to the highest strength (potency) agent, and give "3" to the lowest strength (potency) | Derm-Aid 1% (Hydrocortisone) ointment |  |
|  |  | Elomet (Mometasone furoate) 0.1% cream |  |
|  |  | Dermovate (clobetasol propionate) 0.05% ointment |  |
