# Supplementary material for: Community pharmacists’ counseling practices and patient experiences about topical corticosteroids – an online survey in the Klang Valley, Malaysia
Source: BMC Prim Care. 2022 Oct 15;23:263. doi: 10.1186/s12875-022-01871-z (PMC9569015; doi:10.1186/s12875-022-01871-z)
Supplement: Supplementary file 2 — Supplementary Material 2 [file 12875_2022_1871_MOESM2_ESM.docx]

A questionnaire to study patient and medical characteristics and their perception regarding community pharmacist-provided counseling.

|  | **Item** | **Information** | | **Code** | |
| --- | --- | --- | --- | --- | --- |
| **A. Demographic characteristics** | | | | | |
| **1** | Age (years) |  |  | |  |
| **2** | Gender | Male |  | | 1 |
|  |  | Female |  | | 2 |
| **3** | Ethnicity | Malay |  | | 1 |
|  |  | Chinese |  | | 2 |
|  |  | Indian |  | | 3 |
|  |  | Bumiputera Sabah or Sarawak |  | | 3 |
|  |  | Others |  | | 3 |
| **4** | Pharmacy Location | Selangor |  | | 1 |
|  |  | Kuala Lumpur |  | | 2 |
|  |  | Putrajaya |  | | 2 |
| **5** | Please fill in the name of the cream/ ointment you purchased from the pharmacy |  |  | |  |
| **6** | Was the purchased steroid cream/ ointment for you or for someone else? | For myself |  | | 1 |
|  |  | For someone else |  | | 2 |

| **B. Post-Counselling Checklist** | | | | |
| --- | --- | --- | --- | --- |
| **7** | Please answer "Yes" or "No" for the following questions | | |  |
|  |  | Yes | No |  |
| **a.** | Did your pharmacist inform you that the product is a topical corticosteroid? |  |  |  |
| **b.** | Did your pharmacist explain what skin conditions or diseases where the steroid cream/ ointment should not be used? (Example: not to be used on burns) |  |  |  |
| **c.** | Did your pharmacist explain the strength of the steroid cream/ ointment you purchased? |  |  |  |
| **d.** | Did your pharmacist explain the potential side effects that could occur when using the steroid cream/ ointment? |  |  |  |
| **e.** | Did your pharmacist explain what you should do if you experience side effects from using the steroid cream/ ointment? |  |  |  |
| **f.** | Did your pharmacist explain how to use the steroid cream/ointment for your condition? |  |  |  |
| **g.** | Did your pharmacist explain how often (how many times a day) to use the steroid cream/ ointment in a day for your condition? |  |  |  |
| **h.** | Did your pharmacist explain the duration (how long/ how many days) to use the steroid cream/ ointment for your condition? |  |  |  |
| **i.** | Did your pharmacist inform you how to store and use leftover cream/ ointment after you have completed your treatment? (example: do not use the remaining medication in other conditions at your own discretion) |  |  |  |
